# A Comparison of Reproductive Performances in Young and Old Females: A Case Study on the Atlantic Bluefin Tuna in the Mediterranean Sea

**DOI:** 10.3390/ani11123340

**Published:** 2021-11-23

**Authors:** Luca Marisaldi, Orsola Iorillo, Danilo Basili, Giorgia Gioacchini, Julien Bobe, Violette Thermes, Francesca Maradonna, Oliana Carnevali

**Affiliations:** 1 Department of Life and Environmental Sciences, Università Politecnica delle Marche Via Brecce Bianche, 60131 Ancona, Italy; l.marisaldi@pm.univpm.it (L.M.); orsolaiorillo93@gmail.com (O.I.); d.basili.liv@gmail.com (D.B.); giorgia.gioacchini@univpm.it (G.G.); f.maradonna@univpm.it (F.M.); 2 INRAE Laboratoire de Physiologie et Génomique des Poissons, 35042 Rennes, France; julien.bobe@inrae.fr (J.B.); violette.thermes@inrae.fr (V.T.)

**Keywords:** Atlantic bluefin tuna, miRNA, mir-202, reproduction

## Abstract

**Simple Summary:**

The Atlantic bluefin tuna Thunnus thynnus is a species characterized by complex trans-oceanic migrations linked to size, which rely on the delicate trade-off between somatic growth and reproduction before and during the migratory movements to reach spawning grounds. Therefore, understanding the processes that drive reproduction and elucidating its age-related regulation is essential in the context of sustainable fishery management. In this study, carried out in the Mediterranean Sea, older bluefin tuna females were found to have greater reproductive performances than younger females according to a molecular biology approach (i.e., gene expression), a result that likely mirrors a better physical condition, different habitat usage or migratory behaviour. This result highlights the importance of preserving large females for their major reproductive contribution at a stock level. Furthermore, the gonad-specific mir-202, which belongs to a class of non-coding RNA, called miRNA, that regulate the post-transcription of protein-coding genes, was identified as a potential candidate to play a role in egg quality and quantity (i.e., fecundity) during ovarian maturation through age- or stage-dependent reproductive processes. Overall, the present study contributes to improve the sustainability of the Atlantic bluefin tuna fishery in the Mediterranean Sea.

**Abstract:**

In the Mediterranean Sea, a demographic substructure of the Atlantic bluefin tuna *Thunnus thynnus* has emerged over the last decade, with old and young individuals exhibiting different horizontal movements and spatial–temporal patterns of gonad maturation. In the present study, histology and molecular reproductive markers were integrated with the gonad-specific mir-202 gene expression and ovarian localization to provide a comprehensive picture of the reproductive performances in young and old females and investigate the role played by the mir-202 during gonadal maturation. During the reproductive period, old females (>100 kg; 194.6 ± 33.9 cm straight fork length; 11.3 ± 2.7 years old) were found to have greater reproductive performances than younger females (<80 kg; 139.3 ± 18.8 cm straight fork length; 8.4 ± 1.1 years old) according to gene expression results, suggesting a prolonged spawning season, earlier arrival on spawning grounds and/or better condition in older females. The mir-202-5p showed no global changes; it was abundantly expressed in granulosa cells and faintly present in the ooplasm. On the other hand, the mir-202-3p expression profile reflected levels of oocyte maturation molecular markers (*star*, *lhr*) and both histological and molecular (*casp3*) levels of follicular atresia. Overall, old females exhibited greater reproductive performances than younger females, likely reflecting different reproductive dynamics linked to the physical condition, habitat usage and migratory behaviour. These results highlight the importance of preserving large and old females in the context of fishery management. Finally, the mir-202 appears to be a good candidate to regulate the reproductive output of this species in an autocrine/paracrine manner through either stage- or age-dependent processes.

## 1. Introduction

At the population level, a variety of animal species undertakes seasonal migration to areas that serve as breeding or natal grounds, whose timing is known to vary according to internal signals and environmental factors [1]. Endocrine signals such as sex hormones or glucocorticoids are well-established drivers of observed behaviours in the wild, triggering adaptive responses and shifts in reproductive states [2,3]. In the context of reproduction, the ovary harbours a coordinated network that integrates local and systemic signals, leading to the production of viable eggs and defining the reproductive output [4,5]. In addition to circulating endocrine signals as the gonadotropins, the cross-talk between the follicular cells and the growing oocyte is essential for successful reproduction [6] and local factors such as small non-coding RNAs are emerging as important signals to regulate folliculogenesis [7,8] and to influence the maternal signature of the eggs [9].

The miRNAs are a class of small non-coding RNAs that regulate gene expression through post-transcriptional control of target mRNAs [10] and provide robustness to gene expression networks [11]. Despite being a relatively recent topic, miRNAs have transformed our understanding of the mechanisms of gene regulation in almost all cellular functions, biological processes, life histories and response to environmental stressors [11,12]. So far, the mir-202 has been identified as gonad-specific in several teleost species [13,14,15,16] and such a pattern of expression appears to be conserved, with similar results also reported in mouse [17] and frog [8]. In recent years, new insights have emerged regarding its role in reproduction, highlighting its importance in regulating multiple reproductive processes and relevance as useful molecular marker. Indeed, it was found to regulate the fecundity of the medaka *Oryzias latipes* likely through the control of early steps of follicular development [18]. Furthermore, maternal mir-202-5p was identified as a novel germ plasm-specific marker in the zebrafish *Danio rerio* [13] and was essential for the proper primordial germ cells migration during embryo development [19].

The iconic Atlantic bluefin tuna *Thunnus thynnus* is a highly valuable species characterized by trans-oceanic movements and one of the biggest species inhabiting the pelagic environment [20,21] as it can reach a length of 3.3 m and a weight of 725 kg [22]. In spring, the Atlantic bluefin tuna perform a long migration from feeding grounds in the Northern Atlantic to spawning grounds in the Gulf of Mexico (Western stock) and the Mediterranean Sea (Eastern stock) [20], whose management is based on the commonly accepted division at the 45° meridian which reflects natal homing, although the measure is still debated [23,24,25]. Indeed, a fraction of both small and large spawners from the Eastern stock do not make this long migration because they form a substructure resident in the Mediterranean [26,27] that reproduces in various parts of this sea, which is not entirely compatible with the classic two stocks hypothesis [28]. Part of the fish born in the Mediterranean Sea will leave for the Atlantic at 4–5 months of age (<1 kg) [29], where they will remain until they reach sexual maturity. During those years, they mainly make small migrations within the temperate ocean zone, e.g., area from the Atlantic waters of Morocco [26] to the Bay of Biscay, or even sporadically they may cross the Strait of Gibraltar and enter the Mediterranean Sea [27]. Meanwhile, during the boreal summer, the young bluefin tuna that remained in the Mediterranean Sea are concentrated in the gulfs of Lion and Genoa, and the North Adriatic Sea [30,31,32]. The behaviour of these groups, until they reach the age of sexual maturity, is the same as that of their siblings who went out to the Atlantic. Curiously, all the areas of summer concentration of juveniles (B. of Biscay, G. of Lion, G. of Genoa and North Adriatic) have the same latitude. Although bluefin tuna migrations become greater as the fish get larger, suggesting that movement patterns are linked to the size [20,27,28,29,33,34], young bluefin tunas (1–2 years old; 5–15 kg) can perform transatlantic migrations from New Jersey–Cape Cod area to the Bay of Biscay, and vice versa, as demonstrated by tagging [35,36,37]. The complex highly migratory behaviour of this species represents a significant physiological challenge because of the need to allocate energy for growth, sexual maturation and reproduction as well as to balance environmental conditions [38]. Therefore, the success of this species relies on the delicate trade-off between somatic growth and reproduction before and during the migratory movements to reach spawning grounds.

Certainly, as a species of high commercial interest, good management of the Atlantic bluefin tuna fishery is needed to conserve the species, and the accuracy of stock assessment estimates will be of benefit if a good understanding of the relative contribution of age classes to the reproductive output is achieved [39]. Indeed, mounting evidence is highlighting the importance of old large females in fostering stock productivity, stability and recruitment due to their disproportionally high contribution in terms of reproductive output [40].

In the present study, we sought to test the hypothesis that old females of Atlantic bluefin tuna in the Mediterranean Sea exhibited greater reproductive performances than young females and that the mir-202 could contribute in the regulation of the underlying reproductive processes. Furthermore, reproductively active individuals were compared with non-reproductive individuals to establish a reproductive baseline. The findings of this work will help to better understand the local control of oogenesis by the gonad-specific mir-202 and to elucidate the age-related reproductive output of this highly migratory species that reproduce seasonally.

## 2. Material and Methods

### 2.1. Experimental Design and Sampling Activities

A total of 26 adult ovary samples were collected in 2017–2018. During the reproductive season (*n* = 13, mid-May to late-June) samples were collected in waters south-west of Sardinia in the tuna traps of Carloforte and Portoscuso (Italy), which historically exhibited catches of individuals of age classes from 4 to more than 10 [41]. Then, during the non-reproductive season (*n* = 13, November) additional ovary samples were taken at a tuna farm in Malta to define a reproductive baseline to which compare changes observed in the reproductive season. Furthermore, during sampling procedures, samples of stomach, intestine and liver were also collected. Upon hauling, straight fork length (SFL) and curved fork length (CFL) were recorded to the nearest cm and total body weight was registered to the nearest kg. The range of sizes of weights of sampled specimens was 125–232 cm SFL and 35–257 kg, respectively. The age was estimated using the growth equation in use by the ICCAT Scientific Committee [42]. The biometric data, sampling site and estimated age for each specimen can be found in the Supplementary Materials S1. The samples for molecular biology applications were stored in RNAlater^®^ (Ambion, Austin, TX, USA) at 4 °C and then placed at −20 °C for long-term storage while samples for histological analysis were fixed in a formaldehyde-glutaraldehyde solution (formaldehyde 36.5%, glutaraldehyde 25%, NaH_2_PO_4_, NaOH) and kept at 4 °C. The fish were caught only for commercial purpose and the procedures did not include animal experimentation and ethics approval was not necessary according to the Italian legislation (D.L. 4 of March 2014, n. 26, art. 2).

### 2.2. H&E Histological Processing and Image Analysis

Upon fixation, small ovary pieces of about 3–4 mm were dehydrated through graded ethanol of increasing concentration, cleared in xylene and embedded in paraffin. Sections of 5-µm thick were cut with a microtome Leica RM2125 RTS (Leica Biosystems, Milan, Italy), stained with Mayer’s haematoxylin-eosin and mounted on a glass slide with SafeMount^®^ (Bio-Optica, Milan, Italy). Mounted slides were observed under the Zeiss Aixio Imager M2 microscope and photographed with a high-resolution camera (ZEISS Axiocam 105 colour). The reproductive stages of ABFT females were classified according to [43]. This classification consists of 4 stages, based on the observed histological features, as follows: active non-spawning (ANS), active spawning (AS), inactive mature (IM) and resting (R). The frequency of late vitellogenic oocytes and the rate of α-atresia was calculated according to [44]. The mean diameter of oocytes for each specimen was manually calculated as the mean value of the minor and major axis taken randomly from 30 oocytes cross-sectioned through the nucleus. The image processing and measurements were carried out with Fiji [45].

### 2.3. RNA Extraction and cDNA Synthesis

For each sample, small (<200 bp) and total (>200 bp) RNA were separately extracted using RNAzol RT (Sigma-Aldrich, St. Louis, MI, USA) according to the manufacturer’s instructions with some modifications to enrich the miRNA-containing fraction. The extracted RNA from each sample was diluted in at least 20 μL of RNAse-free water and analysed with the nanophotometer P330 (Implen, München, Germany) to check concentration, 260/230 and 260/280 ratios. The extracted RNA was then stored at −80 °C. Before cDNA synthesis, RNA was treated with the DNase I (Sigma-Aldrich, St. Louis, MI, USA) according to the manufacturer’s instructions. To perform an adequate qPCR for the small-RNA fraction (<200 bp), a total of 2 μg of RNA was polyadenylated with *E. coli* poly(A) polymerase (New England Biolabs, Ipswich, MA, USA) and converted into cDNA with the high-capacity cDNA reverse transcription kit (Applied Biosystem, Waltham, MA, USA) primed with an oligo dT adaptor (Supplementary Materials S2). For the RNA fraction >200 bp, upon DNase I treatment, a total of 2 μg of RNA was converted into cDNA using the high-capacity cDNA reverse transcription kit (Applied Biosystem) following the manufacturer’s protocol.

### 2.4. miRNA Identification

Known teleost mature sequences for mir-202 were retrieved from miRbase (http://www.mirbase.org/; accessed on 1 December 2018) and aligned using Blast with the available genomic resources on NCBI for the Atlantic bluefin tuna (BioProject: PRJNA432036). Upon alignment, the corresponding matching region was extended in opposite directions to obtain the sequence containing the precursor. Then, a similarity search was performed on miRBase to identify the precursor sequence, whose overall features (i.e., minimum free energy, hairpin structure, bulges) were evaluated with the RNAfold web server (http://rna.tbi.univie.ac.at/; accessed on 1 January 2019). The two arms (5p and 3p) were experimentally identified within the obtained precursor sequence through qPCR using primers starting at different positions along the hairpin, guided by a heterologous approach, until amplification, melting curves and gel electrophoresis revealed unambiguous and clear signal. Relative 5p/3p length, complementarity along the precursor and loop size were checked for canonical features of bona fide miRNAs [46].

Candidate miRNAs to normalize the expression levels of the mir-202 were screened according to previous studies [47,48] and identified using either the aforementioned strategy or retrieved from a set of highly conserved miRNA identified in the *Thunnus orientalis* [49]. The expression stability of the candidate reference miRNAs was evaluated with NormFinder [50].

### 2.5. Reproductive Markers

Several reproductive markers were screened from previous studies and obtained from the available molecular resources. Accordingly, primers of the vitellogenin receptor (*vtgR*) were used from [51] and primers of caspase 3 (*casp3*), beclin 1 (*bcn1*), cytoplasmic polyadenylation element binding protein 2 (*cpeb2*), steroidogenic acute regulatory protein (*star*) and ciclin B1 (*ccnb1*) were designed with PrimerBlast [49] using the Atlantic bluefin tuna larval transcriptome [52]. The primers for the luteinizing hormone receptor (*lhr*) were designed on the available sequence on NCBI (GeneBank ID: JX459924.1) using the same methods. The list of primers can be found in the Supplementary Materials S2.

### 2.6. Quantitative RT-PCR

For each miRNA sequence, a specific forward primer spanning the entire mature sequence and a reverse universal primer complementary to the poly(T) adaptor was designed (Supplementary Materials S2). Different universal reverse primers were used to adjust for optimal annealing temperatures. For each primer pair, the optimal annealing temperature was assessed by performing a temperature gradient and the best T_m_ was identified as the temperature at which the most specific amplicon and the lowest C_t_ value were obtained. For quantitative expression analysis of miRNAs, each 10 µL reaction mixture consisted of 2 µL of 1:10 diluted cDNA, 5 µL of SYBR^®^ Green PCR Master Mix (Applied Biosystem), 2.6 µL of RNAse-free water and 0.2 µL of each diluted (1:10) forward and reverse primer. For mRNA, 1 µL of 1:10 diluted cDNA was used and the reaction mixture was adjusted accordingly. The qPCR was performed using the CFX Real-Time PCR Detection System (Bio-Rad, Hercules, CA, USA) with the following cycling conditions: 3′ at 95 °C, 40 cycles of 10′′ at 95 °C, 10′′ at the optimal primer pair annealing temperature, 30′′ at 72 °C and 8′ at 72 °C. The specificity of each primer pairs was assessed by using a dissociation curve performed at the end of the amplification and the qPCR products were run on 2% agarose gel stained with Midori green (Nippon Genetics, Düren, Germany) with the GeneRuler Low Range DNA Ladder (Thermo Scientific, Waltham, MA, USA) as reference. All qPCR reactions were performed in duplicate and C_t_ values were obtained by averaging the values of two technical replicates. The relative expression of mRNA levels was calculated by normalizing target genes with *gapdh*, *β-act* and *18s* according to the principles outlined by [53].

### 2.7. Fluorescent In Situ Hybridization (FISH)

Paraffin-embedded ovaries were sectioned (8 μm thickness) with a microtome (HM355, microm). The anti-sense Locked Nucleic Acid (LNA) oligonucleotide was custom designed and produced by Exiqon A/S to label the miR-202-5p form. A LNA Scramble-miR probe (5′-GTGTAACACGTCTATACGCCCA-3′) was used as a negative control. All LNA probes were double-DIG labelled at both 5′ and 3′ ends. FISH was carried out with the microRNA ISH Buffer Set (FFPE) Hybridization Buffer (ref. 90000, Exiqon), according to the manufacturer’s instructions with some minor modifications. Permeabilization was performed for 7 min at room temperature using proteinase-K (10 μg/mL, P2308 Sigma). LNA probes were used at 20 nM at 53 °C (30 °C below the RNA Tm) for 2.5 h. Samples were then incubated overnight at 4 °C with a rabbit anti-DIG-POD antibody (1:500, ref. 11207733910, Roche). Then, the anti-DIG-POD antibody was detected with the TSA-Cy5 substrate (1:50, TSA PLUS Cy5 kit, NEL 745001KT, Perkin Elmer) for 12 min at room temperature in the dark. Nuclei were stained with DAPI (0.1 μg mL^−1^) for 15 min at room temperature in the dark. All pictures were taken with a SP8 two-photon/confocal hybrid microscope. The acquisitions were imported in Fiji with the Bio-Format plugin and a *z* projection was carried out to obtain the final composite (DAPI + Cy5) image.

### 2.8. Statistical Analysis

Due to the initial and expected heterogeneity of samples from wild-caught individuals, a data mining strategy to explore the dataset and to cluster similar samples was adopted. Upon initial scaling, the Principal Component Analysis (PCA) was performed for clustering purposes using a combination of biometric (SFL, total weight, age), histological (mean follicle size, % atresia, number of late-vitellogenic oocytes) and gene expression (mir-202-3p, mir-202-5p) variables. The expression of the reproductive markers was not included in this initial step instead it was evaluated afterwards to assess the validity of the PCA.

Once the groups were established, the homogeneity of the variance for each variable was checked with the Levene’s test. When such a requirement was not met, the data were log-transformed and the test carried out again to confirm the effectiveness of the transformation. Then, the one-way analysis of variance was performed and if statistically significant differences were detected, the Holm procedure for multiple comparisons was chosen as a post hoc test. When parametric tests could not be used, the Kruskal–Wallis non-parametric test followed by pairwise comparisons using the Wilcoxon rank-sum test was applied. Differences were considered statistically significant if *p*-value < 0.05. The plots and statistical analysis were performed with JASP (JASP Team 2020) and RStudio [54] using the packages ggplot2 [55] and MASS [56].

## 3. Results

### 3.1. Ovarian Localization of mir-202-5p

**Figure 1:**
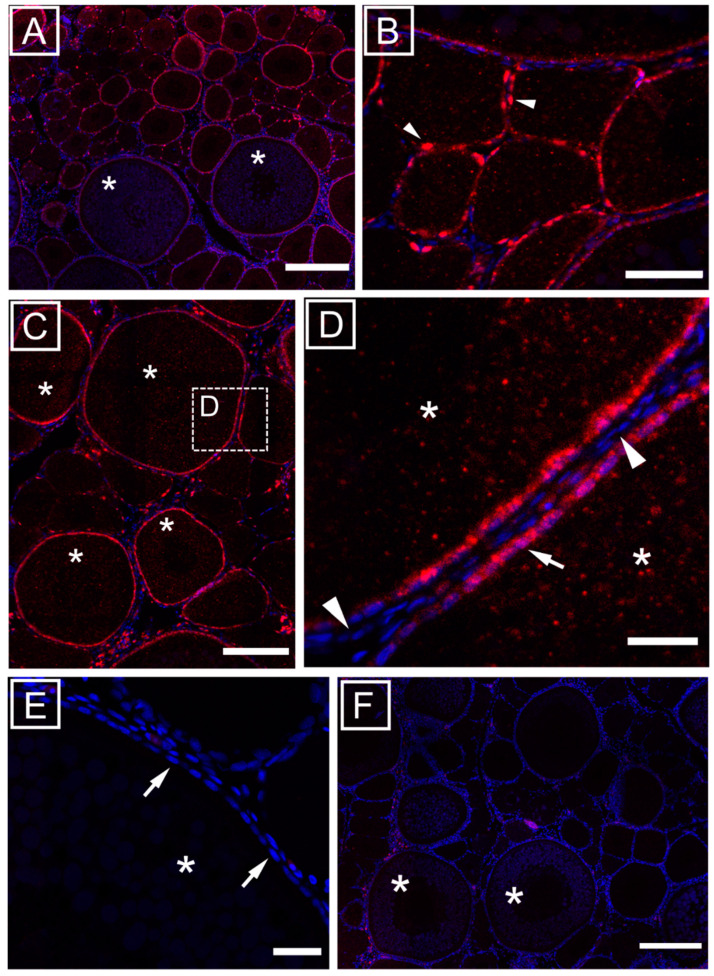
Localization of mir-202-5p expression (red signal) in the ovary revealed with fluorescent in situ hybridization (FISH). Nuclei are stained with DAPI (blue signal). (**A**) View of the ovarian section used for the experiment containing both vitellogenic and previtellogenic oocytes. The mir-202-5p was detected in granulosa cells of previtellogenic ((**B**), arrowhead) and vitellogenic oocytes (**C**) but not in theca cells (**D**). The signal was also faintly detected in the ooplasm (**B**,**D**). Scramble control probe revealed no significant signal above background (**E**,**F**). Asterisks = vitellogenic oocytes, arrows = granulosa cells, arrowheads = theca cells. Scale bars (**A**) 200 µm (**B**) 50 µm (**C**) 100 µm (**D**) 20 µm (**E**) 20 µm (**F**) 200 µm.

The localization of the mir-202-5p was primarily detected in the granulosa cells surrounding previtellogenic and vitellogenic oocytes (Figure 1A–C) but not in theca cells (Figure 1D). A faint, rather homogenous signal was localized within the oocytes in the ooplasm (Figure 1B,D). A progression in the number of granulosa cells could be observed from previtellogenic (perinucleolar, lipid stage) to vitellogenic oocytes and, accordingly, mir-202-5p expression was localized in granulosa cells around previtellogenic oocytes as few spots (Figure 1B), corresponding to a low density of granulosa cells at this stage. To validate the hybridization signal observed with the mir-202-5p probe, the FISH was also carried out using a scramble control probe, revealing no detectable signal above background (Figure 1E,F).

### 3.2. Multivariate Classification Reveals Two Subgroups during the Reproductive Period

**Figure 2:**
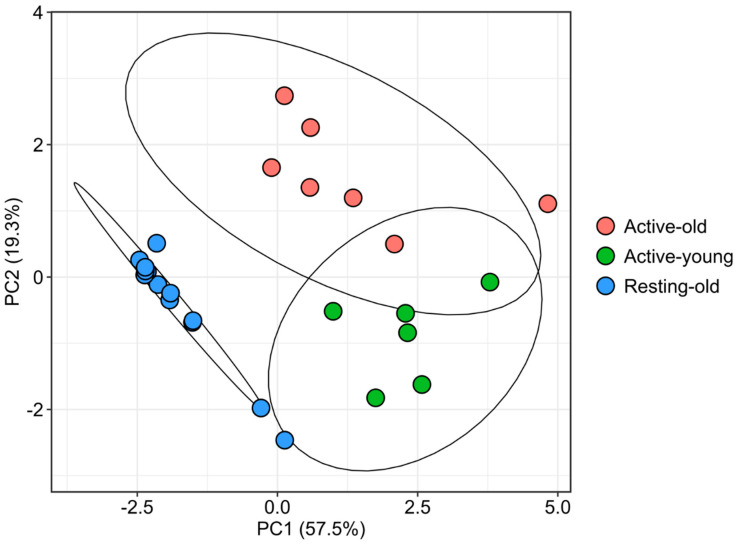
Principal Component Analysis showing the three groups (Active-old, Active-young, Resting-old) identified in the present study. The two subgroups Active-old and Active-young correspond to individuals of the reproductive period and group Resting-old to individuals of the non-reproductive period. The proportion of explained variance along the axes is reported in brackets.

According to the PCA, three groups (Active-old, Active-young, Resting-old) were distinguished, with the first and the second principal components explaining 57.5% and 19.3% of the variance, respectively (Figure 2). The names of the three groups were chosen for a broad classification of age (old-young) and period (Active-Summer; Resting-autumn) and did not represent the histological condition, which was investigated in a separate analysis (see next paragraph). Along the PC1 the two groups belonging to reproductive (Active-old, Active-young) and non-reproductive (Resting-old) periods were well discriminated and, along the PC2, two subgroups within the reproductive period were identified. The group Active-young was composed of smaller and younger individuals, in contrast with the groups Active-old and Resting-old which were represented by bigger individuals of similar size and age (Table 1).

### 3.3. Histological Features of the Ovarian Condition

**Figure 3:**
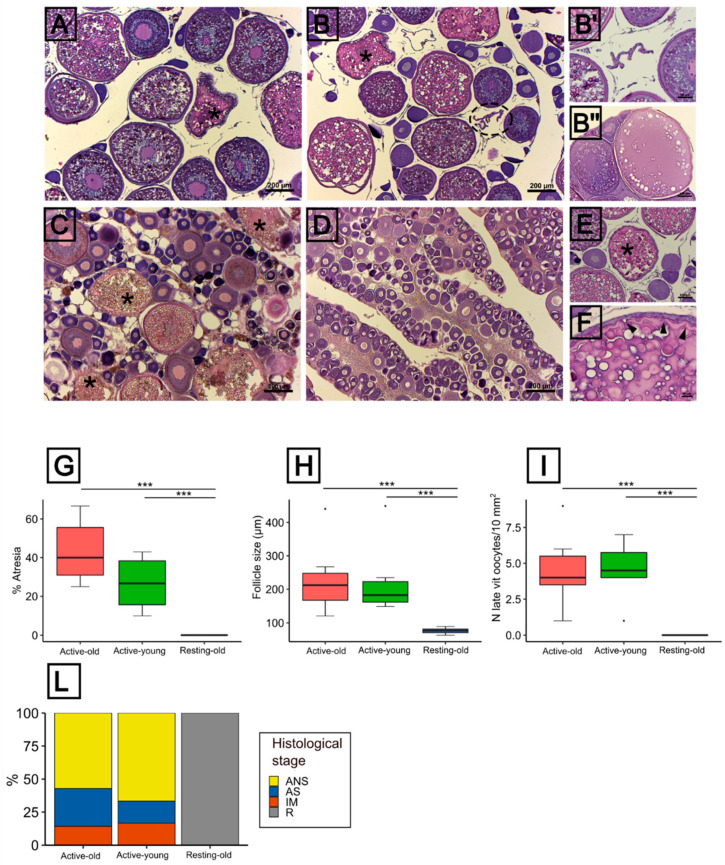
Histological features of the ovaries. (**A**–**D**) Reproductive stages classified according to Schaefer (1998): (**A**) active non-spawning (**B**) active spawning (**C**) inactive mature (**D**) resting; (**B’**) Post-ovulatory follicle (POF); (**B’’**) On the right: maturing oocyte showing yolk coalescence and breakdown of germinal vesicle, on the left: early-mid vitellogenic oocyte; (**E**,**F**) α atretic follicles showing signs of zona radiata breakdown and yolk reabsorption; (**G**) Fraction (%) of atretic follicles; (**H**) Size distribution of oocytes; (**I**) Number of late vitellogenic oocytes/10 mm^2^ ovary section; (**L**) Proportion of histological stages. Asterisks = α atretic follicles; dashed circle = POF; arrowhead = zona radiata breakdown. Statistical significance: *** *p* < 0.001. Histological stage in (**L**): AS = active spawning; ANS = active non-spawning; IM = inactive mature; R = resting.

According to the histological observations, all the 4 stages currently adopted to classify tuna reproductive cycle could be observed (Figure 3A–D). During the reproductive season, the females of the group Active-young contained large late-vitellogenic oocytes in addition to lipid stage, previtellogenic and α atretic oocytes (Figure 3A). The fraction of α atretic oocytes was 26.7 ± 13.8 (Figure 3G), the mean follicle size was 225.7 µm ± 113.5 µm (Figure 3H) and 4.5 ± 2 late vitellogenic oocytes/10 mm^2^ ovary section (Figure 3I). Most of the females in this group were classified as active non spawning (ANS) females, with only two samples classified as active spawning (AS) and inactive mature (IM) females (Figure 3L). The group Active-old was characterized by two females showing signs of imminent spawning (i.e., oocytes exhibiting yolk coalescence, germinal vesicle breakdown) or post-ovulatory follicles (POFs) (Figure 3B,B’,B’’). The rest of the females were classified as ANS, with only one female classified as IM (Figure 3L). The fraction of α atretic oocytes was 43.5 ± 17.1 (Figure 3G), the mean follicle size was 229 µm ± 105.4 µm (Figure 3H) and 4.5 ± 2.5 late vitellogenic oocytes/10 mm^2^ ovary section (Figure 3I). The ovaries of females caught during the non-reproductive period (Resting-old) had lipid stage oocytes as the most advanced stage (Figure 3D), no signs of atresia (Figure 3G) and a mean follicle size of 76.23 µm ± 8.3 µm (Figure 3H). Yet, a small number of atretic follicles (<1%) was observed in certain, though not all, resting ovaries (Supplementary Materials S3) but not included in the analysis according to the method adopted for the calculation, which is based on the percentage ratio of α atretic vitellogenic oocytes on the total number of late vitellogenic oocytes. Atretic follicles (Figure 3E,F) showed typical signs of atresia such as the zona radiata breakdown and yolk resorption (Figure 3F). More advanced stages of atresia, corresponding to the so-called β atresia, were also observed (Supplementary Materials S3).

### 3.4. mir-202 Identification and Expression during Reproductive and Non-Reproductive Periods

Among the reference miRNAs identified in previous studies, a total of three was successfully isolated in the present study: let-7a-5p, let-7e-5p and mir-26a-5p. To evaluate their suitability as references, NormFinder was chosen to evaluate the stability of expression. According to this analysis, the stability value was 0.36 for let-7a-5p, 0.47 for let-7e-5p and 0.56 for mir-26-5p. The best pair (mir-26a-5p, let-7e-5p) showed a stability value of 0.37 and was, therefore, chosen to normalize the expression level of the mir-202 according to the principles outlined by [53]. Instead, in extra-gonadal tissues, the mir-26a-5p was used to normalize expression levels with the 2^−ΔΔct^ method [57] as unspecific amplification was detected for let-7e-5p and let-7a-5p.

**Figure 4:**
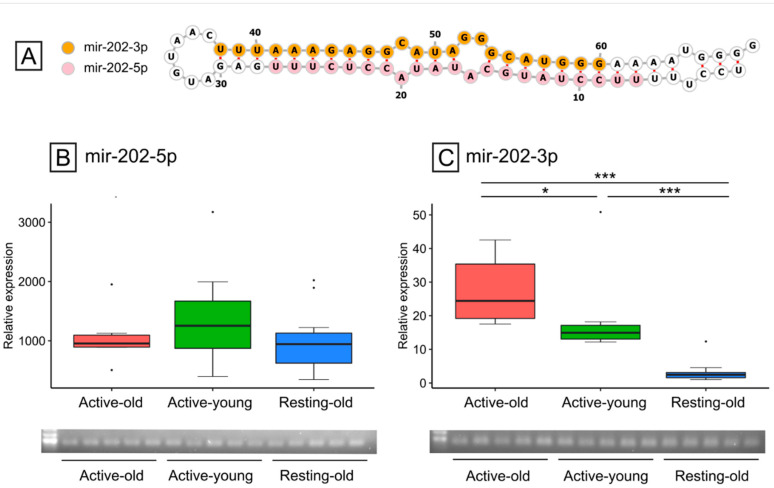
Precursor sequence and expression of mir-202 in the three groups identified. (**A**) Putative hairpin with 5p and 3p arms. (**B**,**C**) Expression of mir-202 in the three groups with the relative 2% agarose gels to further confirm the specificity of the amplification. Statistical significance: *** *p* < 0.001, * *p* < 0.05.

The predicted thermodynamic stability of the putative pre-mir-202 was −31.92 kcal/mol and the overall features of the hairpin resembled those typical of pre-miRNAs (Figure 4A). The expression of mir-202-5p was homogenous among the three groups and, notably, the expression levels during the non-reproductive period were similar to those during the reproductive period (Figure 4B). On the other side, the opposite arm mir-202-3p showed a significant (*p* < 0.05) up-regulation from B to Active-old (Figure 4C). By evaluating the expression in extra-gonadal tissues (stomach, intestine, liver) it was confirmed that mir-202 was mainly expressed in the gonads (Supplementary Materials S4). In extra-gonadal tissues, the mir-202-3p expression was below the detection level.

### 3.5. Gene Expression Analysis of Reproductive Markers

**Figure 5:**
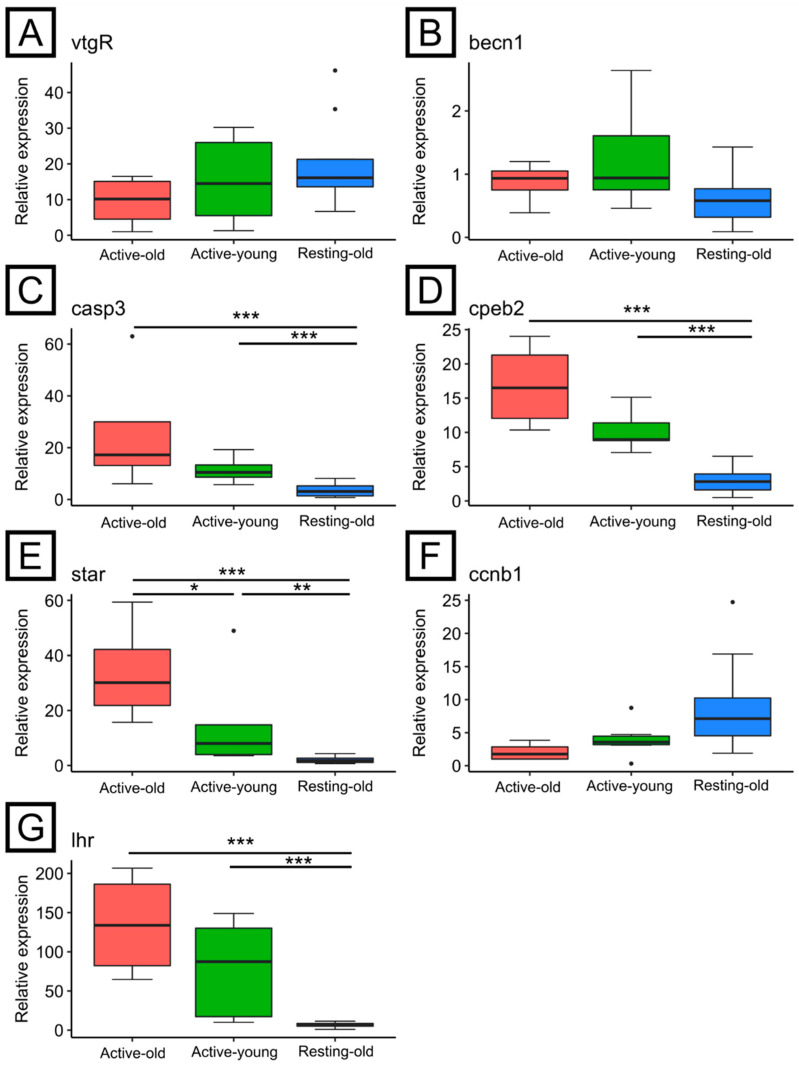
Gene expression profiles of several reproductive markers in the ovary. (**A**) Vitellogenin receptor; (**B**) Beclin 1; (**C**) Caspase 3; (**D**) Cytoplasmic polyadenylation element binding protein 2; (**E**) Steroidogenic acute regulatory protein; (**F**) Ciclin B1; (**G**) Luteinizing hormone receptor. The groups Active-old and Active-young correspond to individuals sampled during the reproductive period while Resting-old group during the non-reproductive period. *** *p* < 0.001, ** *p* < 0.01, * *p* < 0.05.

A general trend, although not statistically significant, of decreased levels of *vtgR* expression was observed from the Resting-old to the Active-old group (Figure 5A) while the expression of *becn1* was homogeneous among groups, with greater variability in the Active-young group (Figure 5B). On the other side, both *casp3* (Figure 5C) and *cpeb2* (Figure 5D) expression levels were higher (*p* < 0.05) in both groups during the reproductive season if compared with the non-reproductive group. The levels of *star* were higher (*p* < 0.05) in the Active-old group than both Active-young and Resting-old groups (Figure 5E), similarly to the levels of *lhr* although in this latter case the difference was statistically significant only between the reproductive groups (Active-young, Active-old) and the Resting-old group (Figure 5G). The levels of *ccnb1* were homogeneous among groups despite an apparent decreasing trend from Resting-old to Active-old (Figure 5F).

## 4. Discussion

In the present study, the majority of females were classified as active non-spawning between mid-May and late-June, in an area corresponding to a migration path to spawning areas (e.g., Balearic Islands, South Tyrrhenian Sea, Sicilian channel) [58,59,60,61,62]. Along the migration route from the Eastern Atlantic Ocean to reach spawning grounds in Western Mediterranean, schools of bluefin tuna exhibit intermediate reproductive traits as found in the Strait of Gibraltar [58,59] and waters southwest of Sardinia [59,60], with the latter area exactly overlapping with the present study. This migration and site fidelity to spawning grounds (i.e., natal homing) appears to be well-established for large migrating adults (>100 kg) by taking into account fishery-dependent catches [61,63] and tagging results [64,65,66,67,68]. However, it is likely that some of the individuals in the present study, especially those from the group Active-young (<80 kg; 8.4 ± 1.1 years old), were resident in the Mediterranean Sea according to the habitat use and migratory behaviour of young/sub-adults found in the area [28,69,70]. Indeed, this observation is also supported by different spatial-temporal patterns of gonad maturation across the Mediterranean Sea, which reflect a more complex demographic substructure and reproductive dynamics [71]. The presence of POFs and maturing oocytes undoubtedly highlights that the bluefin tuna can spawn also in tuna traps, a well-documented phenomenon in transport cages and farms off the eastern coasts of Spain [72,73,74] but irregularly observed in tuna traps in the area investigated [59,60,75]. Future efforts with a focus also on abiotic factors (e.g., water temperature) or surface plankton surveys would be suggested to clarify if it represents a sporadic or regular event and eventually define its relevance in the area.

Both the Active-old and Active-young groups were reproductively active, with similar histological features except for levels of follicular atresia which in general remained around or below 50%, a result supported also by *casp3* mRNA levels. Although levels of the other marker of cell death, *becn1*, were homogeneous among groups, the higher variability observed in the group Active-young might be related to enhanced autophagy, which, upon oocyte failure to recover the required energy, represent the first step of follicular atresia preceding apoptosis along the sequential events that lead to yolk resorption and clearance of follicular cells [76,77]. Between the groups Active-old and Active-young, the former was identified as the most reproductively advanced according to the expression profiles of several molecular markers. Indeed, levels of *star* were higher in Active-old, similarly to *lhr*, although in this latter case they were not statistically different. *Star*, a well-known marker of final oocyte maturation [78], is the key rate-limiting mediator of the steroidogenic response while *lhr* is the central mediator of the systemic LH surge that triggers the last steps of oocyte maturation [79]. Furthermore, levels of *ccnb1* and *vtgR* exhibited an apparent decreasing trend from Resting-old to Active-old. Lower levels of *ccnb1* in the Active-old group might reflect an ongoing translation of the pool of mRNA stored throughout the oogenesis in preparation for the next phase of meiosis resumption via the activation of the M-phase-promoting factor [80]. On the other side, the *vtgR* expression in our study reflected the findings of [51] in the Atlantic bluefin tuna, with higher levels in unyolked oocytes at an early stage of ovarian development well before the spawning season, indicative of temporally uncoupled expression and translation as well as an early preparation for the next spawning season [75]. Interestingly, *cpeb2* exhibited increased expression levels in group Active-old respect to Active-young suggesting a role during the final steps of oocyte maturation to regulate polyadenylation and translation of maternal mRNA, as previously demonstrated for members of the CPEB family [81,82]. It must be noted that, for several genes, the high variability observed in expression levels likely caused a lack of statistical significance among certain comparisons, something that was expected because individuals were wild-caught and/or not under controlled conditions. In summary, the group Active-old, composed by older females than the group Active-young, displayed more advanced reproductive traits at a molecular level, reflecting dynamics that could not yet be observed at a histological scale. Therefore, this reproductive trait highlights greater reproductive performances for older females, suggesting better physical condition, a more protracted duration of the spawning season and/or earlier arrival on breeding grounds for older females, as observed for the southern bluefin tuna *Thunnus maccoyii* [83] and albacore tuna *Thunnus alalunga* [84]. In the Atlantic bluefin tuna, higher fecundity was observed in larger females around the Balearic Islands, although it was linked to their larger ovary size and not affected by the fish length [85]. Nevertheless, their and our results once again highlight the importance of preserving old and large females in a fishery management scenario, confirming their major reproductive contribute at a stock level [40,86].

Currently, understanding the roles of miRNAs in biological systems represents one of the biggest opportunities to clarify processes shaping physiological responses. Here, we showed that the mir-202 is a potential good candidate to regulate the oogenesis through stage- or age-dependent processes. This miRNA was mainly expressed in the gonads and consists of two functional arms differentially regulated, of which the likely dominant arm mir-202-5p is abundantly expressed in the granulosa cells of both vitellogenic and previtellogenic oocytes. A weak expression in the ooplasm would also suggest that this miRNA is maternally accumulated as previously demonstrated in zebrafish developing oocytes [13] and hypothesized in medaka [18]. This implies that offspring viability and survival might be influenced by such a maternal contribution via the successful growth of developing embryos [18,87]. Interestingly, the mir-202-5p showed similar levels of expression during both the reproductive and non-reproductive period, a result in contrast with other fish species in which greater levels of expression were observed as vitellogenesis proceeded [13,14]. However, in zebrafish follicular cells during the transition from small (stage IIIa) to mid (stage IIIb) vitellogenic oocytes, such an increase was also not detected [88], likely due to steady high levels of expression from the cortical alveoli stage onward [89]. The mir-202-5p was proposed, among other functions, to control early development of granulosa cells [18] and to target the transforming growth factor β receptor II (*tgfbr2*) [14], therefore likely to play a role in the progression of follicle development. Since in the Atlantic bluefin tuna the expression levels were quite similar between the two reproductive periods, one of which is characterized by almost null levels of atresia, we would exclude that mir-202-5p mediates follicular atresia in this species. Instead, the mir-202-5p might be involved in early folliculogenesis and/or in the maintenance of the pool of previtellogenic follicles before and during the reproductive season. By regulating the recruitment of previtellogenic oocytes into more advanced stages, this miRNA might control the fecundity and therefore affects the reproductive output of the species, as observed in medaka [18]. On the other hand, the mir-202-3p arm reflected the expression pattern of several genes linked to follicular atresia and oocyte maturation, being more expressed in the most advanced reproductive group. Therefore, it cannot be excluded a role at advanced stages of oogenesis and/or during the progression of follicular atresia. The differential expression and regulation of the 5p and 3p arms represent a well-known process for miRNAs from which mir-202 makes no exception as observed during zebrafish gonadal development [90]. However, a functional approach is needed to fully address such points and provide mechanistic insights of the differential arm usage.

In conclusion, during the reproductive period, older females exhibited greater reproductive performances than the younger ones, highlighting the importance of preserving large and old females in a fishery management scenario. The differences observed between the two age classes can be ascribed to a better condition, a different migratory behaviour, a more prolonged spawning season and/or earlier arrival on spawning grounds for older females. The mir-202 was confirmed as a gonad-specific miRNA, in which the mir-202-3p arm would likely play a role at advanced reproductive stages and/or during follicular atresia while the dominant mir-202-5p arm could be involved in the maintenance and recruitment of the pool of previtellogenic oocytes. Therefore, the mir-202 represents a potential good candidate to regulate the fecundity of the Atlantic bluefin tuna.

## Figures and Tables

**Table 1:** Biometric data of bluefin tuna specimens for each group. Data are shown as mean ± standard deviation. For each variable, different letters correspond to statistically significant differences (*p* < 0.05). SFL: Straight Fork Length.

Group	SFL (cm)	Total Weight (kg)	Age
Active-old (*n* = 7)	194.6 ± 33.9 ^a^	147.1 ± 71.9 ^a^	11.3 ± 2.7 ^a^
Active-young (*n* = 6)	139.3 ± 18.8 ^b^	51.4 ± 28.7 ^b^	8.4 ± 1.1 ^b^
Resting-old (*n* = 13)	210.8 ± 27.4 ^a^	204.8 ± 63.4 ^a^	12.7 ± 2.3 ^a^

## Data Availability

The data presented in this study are available on request from the corresponding author.

## Supplementary Materials

The following are available online at https://www.mdpi.com/article/10.3390/ani11123340/s1, Supplementary Materials S1: Biometrics and sampling information of the specimens used in the study; Supplementary Materials S2: List of primers; Supplementary Materials S3: Additional histology of ovaries during reproductive and non-reproductive periods; Supplementary Materials S4: Expression of mir-202 in gonadal and extra-gonadal tissue.
