# Supplementary material for: A Comparison of Reproductive Performances in Young and Old Females: A Case Study on the Atlantic Bluefin Tuna in the Mediterranean Sea
Source: Animals (Basel). 2021 Nov 23;11(12):3340. doi: 10.3390/ani11123340 (PMC8697984; doi:10.3390/ani11123340)
Supplement: Supplementary file 1 [file animals-11-03340-s001.zip › animals-1471163-supplementary.pdf]

Sampling date	Site	Condition	CFL (cm)	SFL (cm)	Total weight (kg)	Estimated Age
5/6/18	Sardinia	Wild	237	227	241.0	14.3
5/6/18	Sardinia	Wild	229	224	216.0	14
16/5/17	Sardinia	Wild	133	127	35.3	6.7
22/5/17	Sardinia	Wild	172	170	66.5	9.1
5/6/17	Sardinia	Wild	140	138	34.7	7
6/6/17	Sardinia	Wild	143	140	55.8	7.1
6/6/17	Sardinia	Wild	131	130	39.0	6.6
6/6/17	Sardinia	Wild	126	125	35.9	6.3
21/6/18	Sardinia	Wild	213	208	162.4	12.3
24/5/18	Sardinia	Wild	218	211	163.6	12.6
13/6/18	Sardinia	Wild	138	132	45.2	6.7
15/6/18	Sardinia	Wild	181	176	107.8	9.5
28/6/18	Sardinia	Wild	196	190	135.1	10.7
31/10/17	Malta	Farmed	213	204	190.0	11.9
31/10/17	Malta	Farmed	169	163	91.0	8.5
02/11/17	Malta	Farmed	231	221	220.0	13.6
04/11/17	Malta	Farmed	236	226	242.0	14.1
04/11/17	Malta	Farmed	215	206	179.0	12.1
04/11/17	Malta	Farmed	236	226	248.0	14.1
04/11/17	Malta	Farmed	225	215	211.0	13
04/11/17	Malta	Farmed	149	144	58.0	7.3
05/11/17	Malta	Farmed	225	215	212.0	13
09/11/17	Malta	Farmed	238	228	249.0	14.3
09/11/17	Malta	Farmed	241	230	253.0	14.7
09/11/17	Malta	Farmed	241	231	253.5	14.7
09/11/17	Malta	Farmed	243	232	257.0	14.9

Supplementary Materials S1. Biometrics and sampling information of the specimens used in the study

Name	Sequence	Length
mir-202-5p	TTCCTATGCATATACCTCTTT	21
mir-202-3p	TTTAAAGAGGCATAGGGCATGGG	23
let-7a-5p	TGAGGTAGTAGGTTGTATAGTT	22
let-7e-5p	TGAGGTAGTAGATTGAATAGTT	22
mir-26a-5p	TTCAAGTAATCCAGGATAGGCT	22
Oligo dT adapter	GCGAGCACAGAATTAATACGACTCACTAT AGGTTTTTTTTTTTTVN	46
Universal reverse #1	GCGAGCACAGAATTAATACGACTC	24
Universal reverse #2	GCGAGCACAGAATTAATACG	20
Universal reverse #3	GCGAGCACAGAATTAATACGACTCAC	26
Universal reverse #4	GCGAGCACAGAATTAATAC	18

Name	Sequence	Length
casp3 - FW	CTCATGGAGGAACACGCTCA	20
casp3 - RV	TCCAGGTAGGTTGGAGGAGG	20
vtgR - FW	GTGTACCATGTGCTGATCCAG	21
vtgR - RV	TGCTCACATTAGCTTCAGGCC	21
cpeb2 - FW	CAGCAAATCCGGGAAGCAAA	20
cpeb2 - RV	CTGCGTCGGGCAAAAATGAC	20
becn1 - FW	AACCAGATGCGTTACTGCCA	20
becn1 - RV	CACTCCACAGGAACACTGGG	20
lhr - FW	GCTGCCCCTGGTTGGTATAA	20
lhr - RV	ACCGCCAGATAGATCAGCAC	20
star - FW	ATTGTGGCTGCGAATGGAGA	20
star - RV	TGCTGCTCCAACATCACCTC	20
ccnb1 - FW	TGGAGACTTCTGGTTGCGAG	20
ccnb1 - RV	CAACCTCAAGCTGTCGGAGA	20

Supplementary Materials S2. List of primers

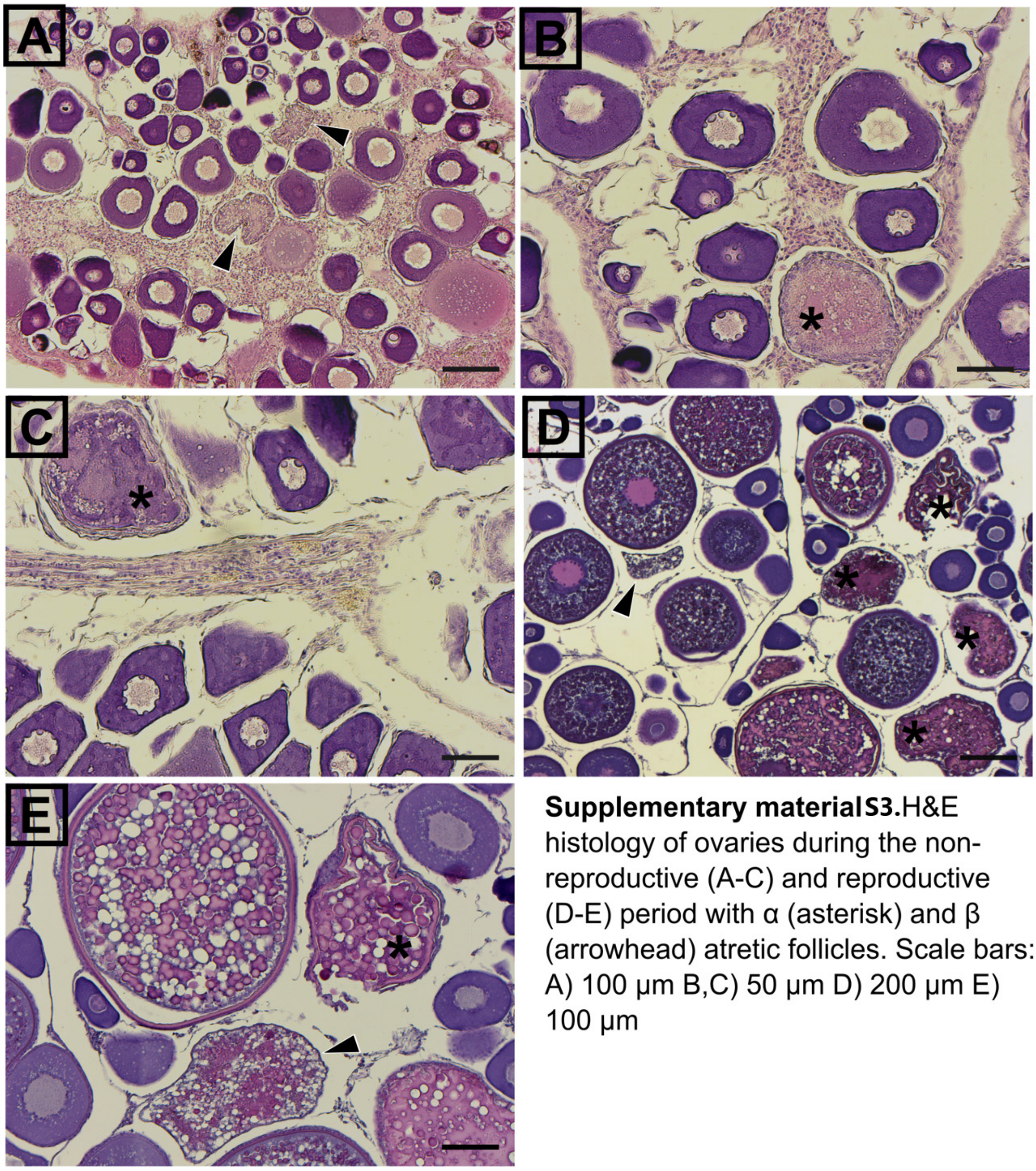

Supplementary material S3. H&E histology of ovaries during the non-reproductive (A-C) and reproductive (D-E) period with α (asterisk) and β (arrowhead) atretic follicles. Scale bars: A) 100 µm B,C) 50 µm D) 200 µm E) 100 µm

Supplementary Materials S3

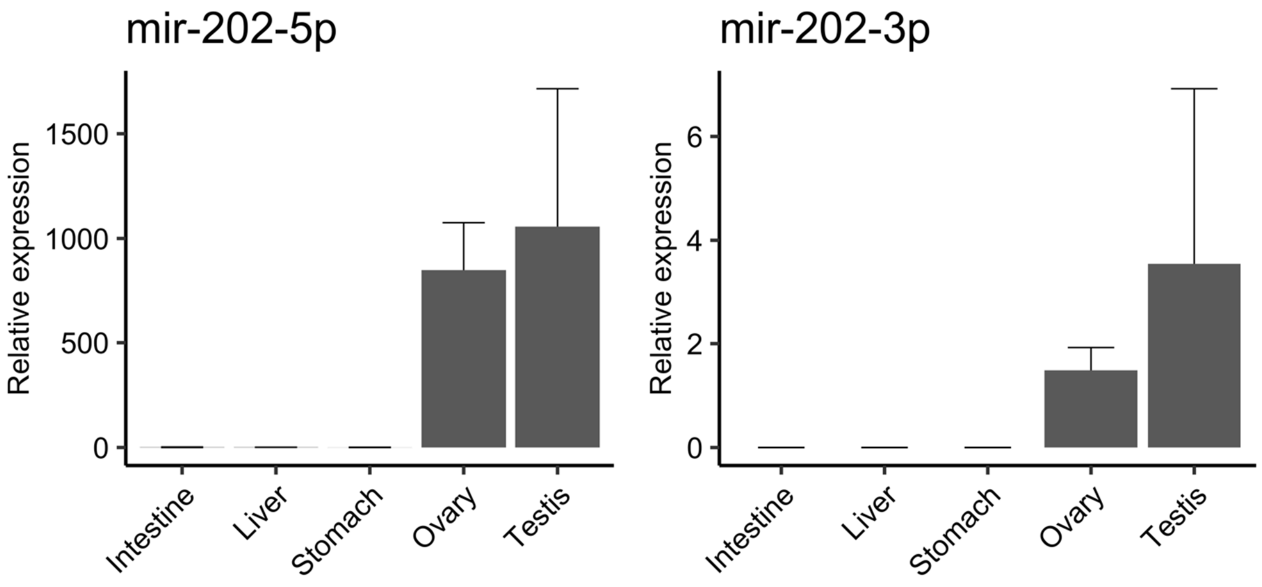

Supplementary Materials S4. Expression of mir-202 in gonadal and extra-gonadal tissue. Each tissue consists of three biological replicates. Bars and error bars corresponds to group mean and standard deviation, respectively.
